# Severe hemolysis and vaso‐occlusive crisis due to COVID‐19 infection in a sickle cell disease patient improved after red blood cell exchange

**DOI:** 10.1002/ccr3.3960

**Published:** 2021-02-22

**Authors:** Lina Okar, Mohamad Rezek, Amna Gameil, Yahya Mulikandayhil, Mohamed A. Yassin

**Affiliations:** ^1^ Department of medical education Hamad Medical Corporation Doha Qatar; ^2^ Department of medical oncology, hematology Section National Center for Cancer Care & Research Hamad Medical Corporation Doha Qatar

**Keywords:** acute chest syndrome, COVID‐19, hemoglobinopathies, hemolysis, red blood cell exchange, Sickle cell disease

## Abstract

Even though most data suggest favorable outcome in patients with SCD and COVID‐19 infection, close monitoring remains essential as acute complication may develop unexpectedly. Offering RBC exchange early in the course of infection might improve prognosis.

## Graphical abstract

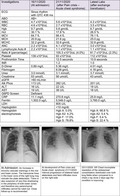

## 1 INTRODUCTION

Sickle Cell disease (SCD) is one of the most common hemoglobinopathies, and it is due to mutation on the beta chain hemoglobin that causes deformity in red blood cells (RBCs) shape. The sickle shape of RBCs affects their function leading to hypoxia and sickle cell crisis including hemolysis when triggers like infections happen.^1^

With the widely spread of Coronavirus infection, special population needs close monitoring as the exact clinical course and prognosis are not well known yet.^2^ It was suggested that patients with SCD might present with vaso‐occlusive crisis (VOCs) or acute chest syndrome (ACS) as first presentation of COVID‐19 infection.^3^ The overlap between ACS and COVID‐19 pneumonia made this an interesting area for research.^4^ However, hemolytic crisis was not very common among previously reported cases.^5^

Management plan for patients with SCD presenting with COVID‐19 infection is not final yet.^6^ However, following the recognized guideline for painful crisis, ACS and hemolysis treatment are essential. Previous studies showed remarkable favorable impact of RBCs exchange in the course of COVID‐19 infection among patients with hemoglobinopathies.^7^ We believe it might prevent deterioration if offered earlier in the course of the disease. Treatment with hydroxyurea (HU) might or might not affect the susceptibility of COVID‐19 infection.^6^ Admission to the hospital is also required even if the cases are asymptomatic or mild because acute complications seem to happen after few days of infection.

Here, we report a case of 48‐year‐old SCD patient compliant with HU who underwent post‐travel screening for COVID‐19 infection and tested positive; admission and close monitoring were offered; he developed painful crisis and severe hemolysis after few days thus needed ICU admission and improved after RBCs exchange.

## 2 CASE PRESENTATION

A 48‐year‐old gentleman came back from a travel abroad and was tested positive for COVID‐19 while screening him as part of post‐travel protocol. Despite being asymptomatic, COVID‐19 PCR from Nasopharyngeal swab was positive with a CT value 15 on 15th November 2020. His past medical history was remarkable for diabetes mellitus type II, on metformin and gliclazide, and sickle cell disease on hydroxyurea 500 mg BID. He had no previous surgeries. He has a history of recurrent pain crisis; most of them were managed in the Emergency department. His last blood transfusion was in 2011. He has hypersplenism and avascular necrosis of the left shoulder, on conservative therapy. His last painful crisis was on 9th October 2020, for which he was managed with IV fluids, pain management, and discharged later in the same day. After testing positive for coronavirus, laboratories (as shown in Table 1) and chest X‐ray (as shown in Table 2) were ordered.

**TABLE 1:** Laboratory results upon admission, deterioration, and discharge. Chest X‐rays

Investigations	16/11/2020 (At admission)	21/11/2020 (after Pain crisis‐Acute chest syndrome)	Latest Laboratories (after exchange transfusion)
ECG	Sinus rhythm with QTC 436 ms		
ABO	AB+		
WBC	4.7 × 10^3^/uL	5.6 × 10^3^/UL	4.3 × 10^3^/uL
RBC	4.6 × 10^6^/uL	2.7 × 10^6^/uL	3.5 × 10^6^/uL
Hgb	9.7 gm/dL	5.8 gm/dL	8.7 gm/dL
Hct	30.1%	17.8%	26.5%
MCV	64.7 fL	66.4 fL	76.1 fL
MCH	20.9 pg	21.6 pg	25.0 pg
MCHC	32.2 gm/dL	32.6 gm/dL	32.8 gm/dL
ANC	2.2 × 10^3^/uL	4.1 × 10^3^/uL	3.0 × 10^3^/uL
Lymphocyte Auto #	2.2 × 10^3^/uL	1.1 × 10^3^/uL	1.1 × 10^3^/uL
Retic # (percentage)		105.3 × 10^3^/uL (4.0%)	81.7 × 10^3^/uL (2.3%)
Platelet	139 × 10^3^/uL	93 × 10^3^/uL	112 × 10^3^/uL
Prothrombin Time		12.5 s	10.9 s
INR		1.2	1.0
D‐Dimer	0.89 mg/L	5.36 mg/L	4.01 mg/L
Fibrinogen		6.09 gm/L	4.18 gm/L
Urea	3.0 mmol/L	3.1 mmol/L	3.7 mmol/L
Creatinine	53 umol/L	56 umol/L	43 umol/L
eGFR	>60 mL/min		
Alk Phos	113 U/L	282 U/L	201 U/L
ALT	16 U/L	22 U/L	19 U/L
AST	19 U/L	38 U/L	18 U/L
G6PD Screen	Deficient		
CRP	11.1 mg/L	270.8 mg/L	25.9 mg/L
Ferritin	1,502.0 ug/L	5,546.0 ug/L	3,785.0 ug/L
LDH		805 U/L	
Haptoglobin		<10 mg/dL	
Hemoglobin electrophoresis		Hgb A: 0.0% Hgb A2: 4.2% Hgb F: 17.0% Hgb S: 78.8%	Hgb A: 66.8% Hgb A2: 5.4% Hgb F: 5.4% Hgb S: 22.4%

**TABLE 2:** Chest X‐ray

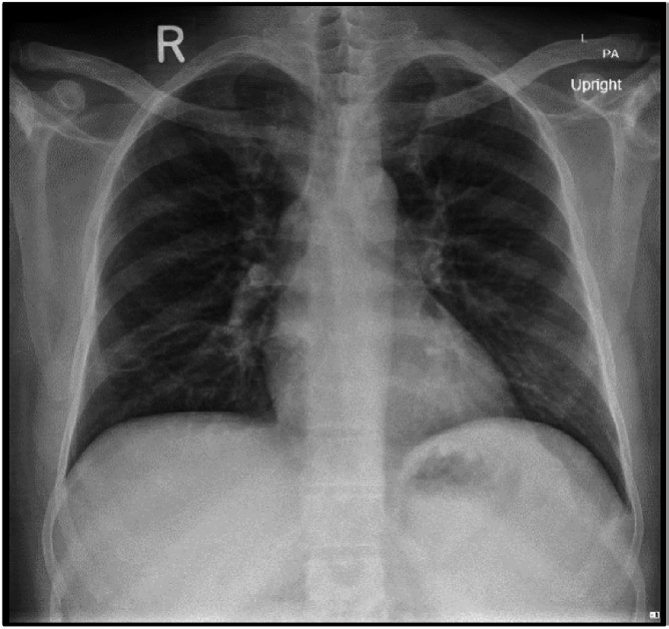	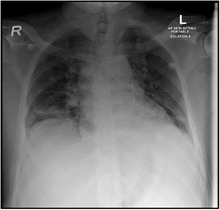	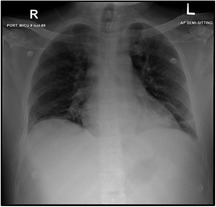
At Admission: An increase in bronchovascular markings in the mid and lower zones. The transverse lines in the lower zone of the right lung may indicate the plate atelectasis. There is also suspicion of very fine shadowing in the periphery of both lower zones, and therefore, any parenchymal infiltrates cannot be ruled out. Close follow‐up is recommended	At development of pain crisis and acute chest syndrome 21/11/2020: Interval progression of bilateral basal atelectasis and faint infiltrates more on the right side	23/11/2020: XR Chest incomplete resolution of the widespread consolidation distributed over both lung fields when compared to a chest X‐ray done 2 days ago the condition is improving

He was started on treatment: Favipiravir + Amoxicillin/Clavulanic acid for COVID‐19 infection pneumonia and plus enoxaparin for deep vein thrombosis (DVT) prophylaxis. On day 4, he started to have intolerable back pain. Laboratories were repeated (Table 1), and the patient was transferred to ICU to manage his pain crisis. Repeated chest X‐ray showed interval progression of bilateral basal atelectasis and faint infiltrates more on the right side, which worsened later. Amoxicillin/Clavulanic acid was changed to Piperacillin/Tazobactam; hemoglobin was low (5.4) with hemolysis picture; and exchange transfusion was done for the patient, with 6 PRBCs. Dexamethasone was also added. The patient became stable after the transfusion and pain management. Another chest X‐ray done 2 days later showed incomplete resolution of the widespread consolidation distributed over both lung fields when compared to a chest X‐ray done 2 days ago his condition was improving. A repeated HbS was 22.3 days later, he was discharged, with instructions about safety netting and home isolation, with resumption of his home medications. Throughout his stay in the hospital, he did not need high oxygen flow nor intubation (the highest oxygen requirement was 1‐2 L nasal cannula for one day)

## 3 DISCUSSION

Coronavirus family has multiple strains, and the latest novel virus caused the coronavirus disease 2019 (COVID‐19) was called severe acute respiratory syndrome coronavirus 2 (SARS‐CoV2).^8^ Data suggested that this new virus acts through specific receptors on the erythrocytes including ACE2, CD147, and CD26 causing pathological interaction with hemoglobin molecule and sometimes resulting in hemolysis.^9^

Sickle cell disease (SCD) is a common inherited hemoglobinopathy. It is due to a single amino acid substitution in the sixth residue of beta globin subunit, and this pathological change results in hemoglobin S (HBS). HBS is an abnormal hemoglobin that causes structure deformity in red blood cells (RBCs) changing it to crescent or sickle shape.^10^

SCD complications are categorized into acute and chronic. The acute presentations are described as time‐dependent which mean there should be high suspension to diagnose them early and offer appropriate evaluation and treatment, thus improving the prognosis. These complications include Acute chest syndrome (ACS), vaso‐occlusive crisis (VOC), acute anemia, hepatobiliary complications, stroke, splenic sequestration, and priapism, whereas chronic complications are pulmonary hypertension, hepatopathy due to iron overload, avascular necrosis, retinopathy, renal disease, and peripheral ulcers.^10^

Hemolytic crisis is defined as drop of more than or equal to 2 g/dL from the baseline patient's hemoglobin value. Acute anemia might present as a single presentation or in accordance with splenomegaly or hepatomegaly.^10^ However, when aplastic crisis diagnosis is made it is essential to rule out any possibility of delayed hemolytic transfusion reaction as more cautions are required if RBCs transfusion is the plan.^1^

ACS is the most cause of hospital admission and mortality among SCD patients. The trigger for this condition is often infection, mostly bacterial and sometimes viral. Both ACS and COVID‐19 pneumonia share similar clinical features such as fever, cough, shortness of breath, and chest pain. Evidence from previous studies suggests that SARS‐CoV2 could trigger ACS.^11^ Due to this overlap between ACS and pneumonia, it is important to recognize the effect of COVID‐19 in SCD patients.^7^

Generally, patients with chronic comorbidities like diabetes, hypertension, lung diseases, and cardiovascular diseases are at increased risk of COVID‐19 complications. Noticeably, patients suffering from SCD have a lot of complications as mentioned before. In the presence of this multi‐organ dysfunction,COVID‐19 infection may trigger ACS or VOC.^12^ Also, the abnormality in endothelial and procoagulants increases the risk of thromboembolic events, thus providing prophylactic anticoagulants is essential.^2^

A recent retrospective study conducted in Bahrain reviewed 387 patients with SCD screened for COVID‐19 infection, 6 patients had COVID‐19 infection, all patients had favorable outcome, 3 patients were asymptomatic, and 3 had mild to moderate symptoms. The study concluded that there is no difference among patients with SCD and non‐SCD patients in terms of COVID‐19 clinical course, viral clearance, and infection rate. As well, no remarkable increased risk for SCD crisis.^9^ In our case the patient had asymptomatic presentation then developed acute painful episode with severe hemolysis. A case series of 10 patients with SCD infected with COVID‐19 in UK also showed a good prognosis as 9 out of 10 patients recovered fully. Interestingly, 7 patients received blood transfusion (4 RBCs exchange and 3 simple transfusions). It is worthy to notice that one patient in this series died and she did not receive blood transfusion as she had delayed hemolytic transfusion reaction as contraindication.^13^ Another case series for 4 patients with SCD and COVID‐19 infection showed that all patient presented with VOCs and had mild course, and only one patient deteriorated and needed intensive care unit (ICU) admission where the patient received RBCs exchange and improved remarkably. This case series suggests good prognosis of COVID‐19 infection and emphasize on the importance of early RBCs exchange. However, none of the four patients mentioned in this case had acute hemolytic anemia.^3^ Nino Balanchivadze et al published one center study that retrospectively reviewed 24 patients with SCD anemia or trait infected with COVID‐19, 4 patients had SCD, and none of the included patients was taking hydroxyurea (HU). The author concluded that patients with SCD generally had unremarkable course of disease, low possibility of ICU admission and intubation, but hospitalization period might be longer.^6^ It has been hypothesized that COVID‐19 infection in patients with SCD precipitates hemolysis because it affects hemoglobin beta chain, whereas others suggested that patients with Thalassemia and SCD have immunity against COVID‐19 infection due to the defect in hemoglobin beta chain^9,14,15^ The physiology behind this hypothesis as published in one paper is that viral proteins like ORF1ab, ORF10, and ORF3a might cause porphyrin‐associated iron molecule in heme to separate, and this complex is located in the beta 1 chain, resulting in less amount of oxygen being carried by the hemoglobin in the blood stream.^15^ Patients with SCD as mentioned have mutation in beta chain hemoglobin, whether this plays a role in the protection against COVID‐19 infection or not is still an area of research.

Red blood cell exchange is one way to treat severe ACS, a previous data suggested its use in the case of severe COVID‐19 pneumonia overlapping with ACS in patients with SCD. Moreover, it was noticed that whenever RBCs exchange offered earlier, the prognosis and clinic course were better^7,3^ In our case, the patient improved after the red blood cell exchange and we suggest that it might play a role in the prevention of painful episode and hemolysis crisis progression if offered earlier in the course of infection.

While RBCs exchange forms an important component in the acute management plan of SCD crisis, hydroxyurea is the mainstone in the maintenance therapy. However, it is not recognized yet if compliance with this therapy affects the presentation of COVID‐19, data in this regard are conflicting, one retrospective study screened 40 patients with SCD for coronavirus infection, only 24 were positive, and all of them were not on HU, thus the authors suggested that HU in treatment might have a protective effect against the virus. This was not the case in our patients as he was on long‐term HU treatment and still had moderate course of illness.^6^

Admission of Patients with SCD and COVID‐19 infection is essential even if they are asymptomatic as they can develop acute painful episodes and hemolytic crisis later in the course of infection like our reported case. Although no final guideline is available now for the treatment of SCD patients with COVID‐19 infection, we believe that offering RBCs exchange is an essential part of managing those populations and we recommend offering it as earlier as possible to avoid deterioration. Finally, the role of HU in the clinical course of coronavirus infection is still not defined and forms an area for further research.

## 4 CONCLUSION

Even though previous studies suggested favorable outcome for COVID‐19 infection among patients with hemoglobinopathies, SCD patients remain at risk of acute deterioration even in the absence of any COVID‐19 characteristic symptoms. Thus, close monitoring is necessary. ACS was mentioned before as a presentation of COVID‐19 infection in those patients, yet acute hemolysis crisis should also be in mind. Offering RBCs exchange and admitting patients to the ICU until they are stable plays a role in improving prognosis and the earlier this is done the better the outcome. We believe that more studies are needed to form a clear guideline regarding RBCs exchange role and time of administration and the role of HU compliance on the course of infection.

## CONFLICT OF INTEREST

The authors report no conflicts of interest.

## AUTHORS’ CONTRIBUTION

LO and MR: wrote the manuscript. AG, YM, and MAY: reviewed the literature and manuscript.

## ETHICAL APPROVAL

Consent was obtained from the patients.

## Data Availability

All data related to this article are available upon request.
